# Evaluating the Efficacy of CPS, HEART and TIMI Score in Emergency Department Patients with Non-Traumatic Chest Pain: A Pilot Study

**DOI:** 10.3390/medsci14010151

**Published:** 2026-03-19

**Authors:** Pietro Pozzessere, Mattia Di Lauro, Francesco Incantalupo, Alessandro Cinquantasei, Stefano Palazzo, Mario Erminio Lepera, Antonella Pistone, Sandra De Matteis, Marco Matteo Ciccone, Vincenzo Brescia, Roberto Lovero, Marcello Albanesi, Angela Pia Cazzolla

**Affiliations:** 1 Department of Emergency Medicine, University Hospital of Bari, 70124 Bari, Italy; pieropozzessere66@gmail.com (P.P.);; 2 “M. Albanesi” Allergy and Immunology Unit, 70124 Bari, Italy; 3 Faculty Medicina and Surgery, University of Bari “Aldo Moro”, 70121 Bari, Italy; 4 The Allergist, 70124 Bari, Italy; 5 Department of Engineering and Science, Universitas Mercatorum, 00186 Rome, Italy; 6 University Cardiologic Unit, Interdisciplinary Department of Medicine, Polyclinic University Hospital, 70120 Bari, Italy; 7 Clinical Pathology Unit, AOU Policlinico Consorziale di Bari-Ospedale Giovanni XXIII, 70124 Bari, Italy; 8 Allergolys, 75000 Paris, France; 9 Department of Medicine and Surgery, University LUM ‘Giuseppe Degennaro’, Casamassima, 70010 Bari, Italy

**Keywords:** chest pain, emergency department, risk stratification, HEART score, TIMI risk Score, Chest Pain Score (CPS), Acute Coronary Syndrome (ACS), clinical scores, triage, cardiovascular risk

## Abstract

Background and Aim: The correct identification of patients presenting with chest pain and the stratification of their risk for major adverse cardiovascular events (MACE) is essential. The aim of this study was to evaluate subjects who came to the ED for chest pain through the chest pain score, the HEART score and the TIMI risk score in order to assess their validity and prognostic accuracy and to compare their performance. Methods: Patients included in the study met the following criteria: age ≥18 years, reported atraumatic chest pain, and consent to participate in the clinical study. Subsequently, the final scores were calculated based on the information collected and a follow-up was performed to assess the occurrence of adverse cardiovascular events (MACEs) at 30 days. The MACEs considered were a composite endpoint of STEMI or NSTEMI myocardial infarction, positive coronary angiography for critical lesions, percutaneous coronary angioplasty, coronary artery bypass grafting, and death. Results: A total of 102 patients were included in the study sample, divided into 76 patients who did not develop MACEs and 26 patients who experienced MACEs. The AUC values of the ROC curves of the chest pain score, HEART score and TIMI risk score were 0.8312, 0.9757 and 0.9378 respectively. Conclusions: All three scores examined were considered excellent tools to predict the onset of MACEs in patients with chest pain at different points of clinical management, although the HEART score outperformed both the chest pain score and the TIMI risk score in terms of prognostic accuracy.

## 1. Introduction

Chest pain is one of the main presenting symptoms in the emergency department (ED), as demonstrated by the more than 7 million ED visits recorded in the United States in 2020, making it the second most common cause of admission [1]. One of the greatest challenges faced by healthcare professionals in the ED—and beyond—is the rapid differentiation between serious, potentially cardiac causes and benign, non-cardiac conditions [2,3].

Potentially life-threatening causes include acute coronary syndrome (ACS), which accounts for up to 10% of acute chest pain diagnoses [4], with one-year mortality rates of 18% for men and 23% for women over 40 years of age following an initial diagnosis of acute myocardial infarction (AMI) [5]. In addition, it is estimated that 1.2 million people in the United States experience an AMI each year, and approximately 7% of hospitalized patients die [6,7,8].

It should also be noted that a proportion of patients with AMI—ranging from 0.9% to 2.1%—are unfortunately not diagnosed during their initial evaluation [6,7,8,9].

Given these data, the objectives of an emergency department are essentially twofold. The first is to safely discharge low-risk patients with chest pain as early as possible, in order to reduce overcrowding and healthcare costs [10]. The second is the rapid identification of high-risk patients who may benefit from immediate diagnostic and/or therapeutic interventions, thereby reducing missed diagnoses and mortality [3].

To achieve these goals, ED healthcare providers must be able to quickly classify patients and stratify them into different risk categories. To support this process, several clinical scoring systems have been developed. These scores, calculated by clinicians, help estimate the risk of adverse events and guide patient management [3].

The aim of this study was to evaluate patients presenting to the ED with chest pain using the chest pain score (CPS), the HEART score, and the TIMI risk score, in order to assess their validity, prognostic reliability, and comparative performance.

## 2. Material and Methods

The prospective observational study was carried out at the emergency department of the University Hospital of Bari between 20 May 2024 and 21 December 2024.

Candidate patients for the study were selected based on the following inclusion criteria: age ≥18 years, reported atraumatic chest pain, and consent to participate in the clinical study.

The study received approval from the ethical committee (nr. 6698: 11/2/2021) of the Policlinic of Bari, in compliance with the Helsinki Declaration. Each patient or their legal representative provided consent for the data to be processed.

At the time of admission to the emergency department, patients were initially evaluated at triage, assigned a color code based on the clinical condition presented and subsequently examined by the doctor in relation to the color code.

Therefore, the subjects who complied with the conditions of admission and signed the informed consent were enrolled, and their personal information was collected with the help of a data collection form. As this was a real-life, non-interventional observational study conducted entirely within the framework of standard clinical care, without any procedures or treatments deviating from national or international guidelines, the local ethics committee granted a formal waiver of informed consent.

The data collection form included personal information, color code, vital and blood biochemical parameters, past medical history and data relating to the founding elements of the risk scores considered.

For each patient, the chest pain score, the HEART score and the TIMI risk score were calculated and the onset of MACEs (major adverse cardiovascular events) within 30 days of admission to the emergency department was assessed via telephone call.

The chest pain score (CPS) evaluates key characteristics of chest pain, including location, quality, radiation, intensity, modifying factors, and associated symptoms [11]. A CPS < 4 indicates a very low probability of coronary artery disease, whereas a CPS ≥ 4 identifies a low-to-intermediate or high probability [12].

The HEART score is based on the assessment of medical history, ECG findings, age, cardiovascular risk factors, and initial troponin levels. Each parameter is assigned a value from 0 to 2, for a total score ranging from 0 to 10. Three risk categories are defined: low risk (0–3), intermediate risk (4–6), and high risk (7–10) for major adverse cardiovascular events [13,14].

The TIMI risk score consists of seven components: age ≥65 years, ≥3 cardiovascular risk factors, known coronary artery stenosis ≥50%, use of acetylsalicylic acid in the previous 7 days, severe angina with ≥2 episodes in the last 24 h, ST segment deviation ≥0.5 mm on ECG, and elevated cardiac biomarkers. Each component is assigned a value of 0 or 1, resulting in a total score between 0 and 7. Scores of 0–2 indicate low risk, 3–5 intermediate risk, and 6–7 high risk of adverse cardiovascular events [15,16,17].

The MACEs considered were a composite endpoint of STEMI or NSTEMI myocardial infarction, positive coronary angiography for critical lesions, percutaneous coronary angioplasty, coronary artery bypass grafting, and death.

The troponin I hs (hs cTnI) test (analytical sensitivity of 0.5 pg/mL, linear range of 0.1–25,000 pg/mL, intra-assay variation of 3.7%, inter-assay variation of 6.6%) was performed with a chemiluminescence assay using Siemens ATELLICA (SIEMENS, Munich, Germany).

### Statistical Analysis

Patients were divided into two groups: those with MACEs and those without MACEs. Medians of continuous variables were described as mean ± standard deviation standard compared using the Mann–Whitney U test. Categorical variables were expressed as n (%) and compared using Fisher’s exact test. Statistical significance was set at *p* < 0.05.

Spearman’s rank correlation coefficient was calculated to assess the correlation between different parameters. An rs value of +1 or −1 was considered indicative of a perfect association, whereas an rs value of 0 indicated no association. The results were reported in tables.

Receiver operating characteristic (ROC) curves were used to derive the sensitivity and specificity of the indices under study. The following classification of AUC values was adopted: 0.9–1.0 excellent accuracy, 0.8–0.9 very good, 0.7–0.8 good, 0.6–0.7 sufficient, 0.5–0.6 poor, and <0.5 useless [18]. A *p*-value threshold of 5% was applied to all statistical tests.

The comparison between AUCs was then validated using DeLong’s test.

To identify the association between individual clinical and laboratory variables and the risk of 30-day MACEs, a univariate analysis was performed using binary logistic regression (Logit model) with parameter estimation performed using maximum likelihood estimation. The results are expressed as odds ratios (ORs) with 95% confidence intervals (95% CIs) and *p*-values associated with the Wald test, estimated individually, modeling the probability of MACEs as a function of each variable (continuous or categorical), rather than starting from group medians or means

To identify independent predictors of 30-day MACEs, single-predictor logistic regression models were constructed separately for total HEART, TIMI and CPS, excluding the individual variables that constitute their components to avoid redundancy and collinearity.

Model performance was evaluated using adjusted odds ratio (OR) with 95% CI, Area Under the Curve (AUC), and Akaike Information Criterion (AIC). AUCs were compared using the DeLong test.

Finally, to identify independent predictors of 30-day MACEs, a multivariate binary logistic regression analysis was conducted, including in the model all variables that were significant in the univariate analysis and with clinical relevance. To improve the clinical interpretability and numerical stability of the model, some variables were scaled: age was expressed per 10-year increase; clinical scores per 1-point increase. Due to the highly skewed distribution of hs-troponin values, these were transformed using log_10_.

Summary tables were produced to highlight variations in the concentrations of the different markers used and the results of the statistical tests.

All statistical analyses were performed using MedCalc version 9.2.0.2 (MedCalc Software, Ostend, Belgium).

## 3. Results

The total number of patients enrolled in the prospective study over the period from 20 May 2024 to 21 December 2024 was 108 subjects, of whom 2 were excluded from the study because the initial reported symptoms of chest pain were unmatched at the time of medical re-evaluation and 4 were lost at follow-up. As a result, the actual number of patients recruited for the study was 102 people.

The 102 patients in the sample included 32 women (31.4% of the total) and 70 men (68.6% of the total) with a mean age of 59.4 years and a standard deviation of ±15.2 years.

On the other hand, evaluating the main cardiovascular outcomes of MACEs at 30 days, it was found that of the 102 patients enrolled, 26 developed MACEs (25.5% of the sample); more precisely, 23 patients had a positive coronary angiography for critical lesions, of which 21 received coronary revascularization subsequently while 2 did not benefit from revascularization surgery. Of the 21 patients (20.6% of the sample) who underwent revascularization, 17 received percutaneous coronary intervention (PCI) and 4 patients underwent coronary artery bypass graft (CABG).

A total of 16 patients (15.7% of the sample) developed acute coronary syndrome (ACS), including 11 diagnoses of NSTEMI and 5 diagnoses of STEMI.

Finally, three patients (2.9% of the sample) died during their hospital stay or at follow-up.

The main characteristics of the patients are expressed in Table 1.

The comparison between patients who developed major adverse cardiovascular events (MACE group) and those who did not (non-MACE group) revealed significant differences in the main clinical scores and troponin values, suggesting a more severe cardiovascular risk profile in the MACE group. The blood concentrations of high-sensitivity cardiac troponin (hs-cTnI) measured at the different sampling times (time 0, time 1 (3 h), and time 2 (6 h)) were evaluated in relation to the three clinical scores under investigation in both the non-MACE and MACE groups.

**Figure 1:**
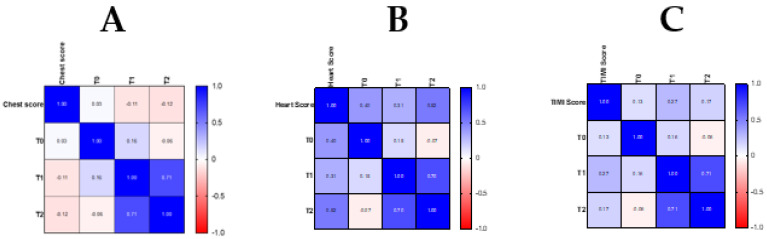
Correlation matrix between CPS and troponin levels at T0, T1, and T2 in the MACE group (**A**). Correlation matrix between HEART score and troponin levels at T0, T1, and T2 in the MACE group (**B**). Correlation matrix between TIMI risk score and troponin levels at T0, T1, and T2 in the MACE group (**C**).

The data of the three scores evaluated together with the hs-cTnI measurements performed at T0, T1 and T2 were entered as statistical variables in a correlation matrix to determine the existence of a possible relationship between them (Figure 1A–C).

The blood values of high-sensitivity cardiac troponin (hs-cTnI) taken at the different sampling times (time 0, time 1 and time 2) were considered in relation to the three clinical scores under study in the two groups of non-MACE and MACE patients

**Figure 2:**
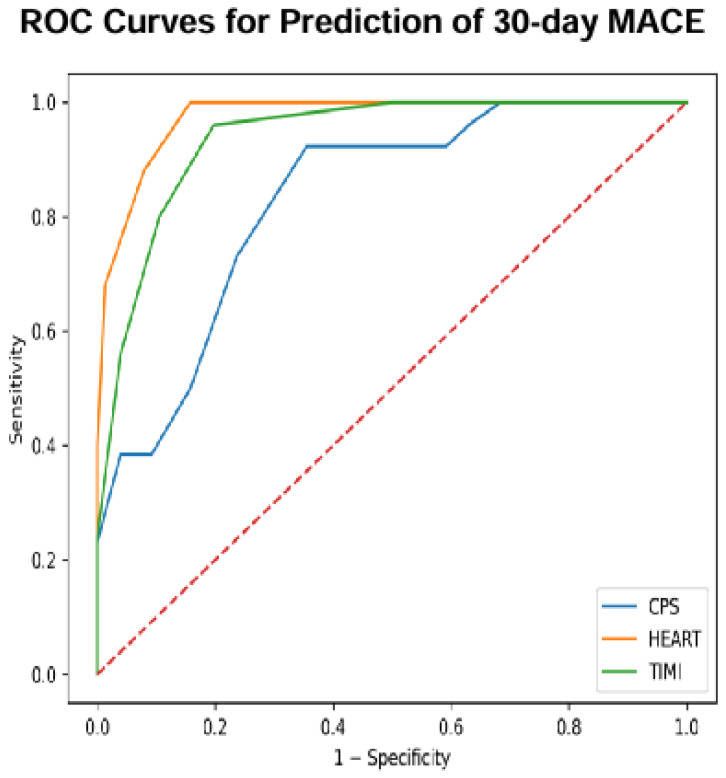
Multiple-comparison ROC curves of the score for 30-day MACE.

Finally, the ROC (receiver operating characteristic) curves of the three scores considered in the study were made to perform a comparison. Multiple-comparison ROC curves showed the three scores considered in this study (Figure 2).

The AUC values of the ROC curves for the three scores, all with *p* < 0.0001, are reported in Table 2.

The ROC curve analysis showed good discriminative ability of the total chest pain score in predicting 30-day MACEs (AUC 0.83). However, its performance was inferior to that of the composite clinical scores. The TIMI risk score demonstrated excellent predictive capacity (AUC 0.94), while the HEART score showed the best discriminative performance, with an AUC of 0.96.

The comparison of AUCs using DeLong’s test revealed statistically significant differences among HEART, TIMI, and CPS (Table 3).

The comparison of ROC curves using DeLong’s test showed that both HEART and TIMI performed significantly better than CPS. No statistically significant difference was observed between HEART and TIMI, indicating comparable discriminative performance.

In the univariate analysis, only the composite clinical scores (CPS, HEART, and TIMI), hs-cTnI at all three time points, and age were significantly associated with the outcome. Given that hs-cTnI is a key component of these scores, its strong association with 30-day MACEs is consistent with its established prognostic relevance (Table 4).

The score variables were described using median (IQR) due to their non-Gaussian distribution, but were modeled as continuous variables in the logistic regression to preserve statistical power and clinical interpretability. The same approach was applied to troponin variables, which were subsequently log-transformed to avoid distortions and ensure clinically interpretable estimates.

The three scores share several conceptual components; we examined their relationships. As expected, HEART and TIMI showed a substantial correlation (r = 0.872), reflecting their overlapping clinical structure. This correlation was considered when interpreting comparative model performance.

To identify independent predictors of 30-day MACEs, we constructed separate single-predictor logistic regression models for total HEART, TIMI, and CPS, excluding additional covariates already embedded within each score to avoid redundancy and structural collinearity These analyses confirmed that all three scores were significantly associated with 30-day MACEs, with HEART and TIMI showing the highest discriminative performance.

Lower AIC values indicate a better balance between model fit and parsimony, whereas higher AUC values reflect superior discriminative ability. AUCs were statistically compared using DeLong’s test.

Although the HEART model showed the highest numerical AUC and the lowest AIC, DeLong’s test did not reveal statistically significant differences between HEART and TIMI. Therefore, HEART cannot be considered superior to TIMI; instead, the two scores demonstrated comparable discriminative performance.

Given the substantial correlation between HEART and TIMI (r = 0.872), and considering that VIF assesses collinearity rather than incremental predictive value, conclusions regarding added performance cannot be based on VIF. For this reason, we formally evaluated whether adding CPS to HEART improved predictive ability using appropriate model comparison metrics.

Comparison between the HEART-only and HEART + CPS models (Table 5) showed nearly identical AUC values and a higher AIC for the combined model. The Likelihood Ratio Test (χ^2^ = 0.59, df = 1, *p* = 0.444) confirmed that adding CPS to HEART does not significantly improve discrimination or model fit.

## 4. Discussion

In our prospective and pilot study, the performance of the CPS was compared for the first time with those of the HEART and TIMI scores in predicting the occurrence of 30-day MACEs in patients presenting to the emergency department with non-traumatic chest pain and without STEMI on the initial ECG.

### 4.1. Chest Pain Score

The CPS was administered during a post-triage interview. The score was first introduced by Geleijnse in 2000 [19]. Its administration time is approximately 2 min.

For the calculation of the chest pain score, a cut-off of 4 was established in the prospective study by Conti et al. [13]. A CPS < 4 is indicative of a very low probability of coronary artery disease, as opposed to a CPS ≥ 4, which identifies a low–intermediate or high probability [20,21].

The chest pain score calculated for the MACE group showed that 100% of patients belonging to this group (26 patients) had a result ≥4 with no patients in this group having a score <4 (0%). However, the specificity of CPS is reduced if we consider that in the non-MACE group 52 patients (68.4%) achieved a score ≥4 and the remaining 24 patients (31.6%) achieved a score <4.

From these results, in accordance with the scientific evidence available in the literature, it can be deduced that the chest pain score is an excellent clinical tool to further stratify the group at low risk of developing cardiovascular events [8,22,23]. Chest pain score is a tool with good discriminative ability and indicates that the clinical assessment of chest pain alone has substantial value, although its performance is slightly inferior to that of composite risk scores. Its use at triage by nursing staff becomes important for assigning an appropriate severity code and for the initial risk stratification of patients presenting with chest pain.

### 4.2. HEART Score

The ability of the HEART score to correlate with the severity of coronary artery disease has been demonstrated in recent studies and reviews [24,25].

The use of hs-cTnI was expected to improve the prediction of major cardiovascular events, but this depends on the different reagents available on the market and on the various manufacturing companies [26,27,28].

The calculation of the HEART score allowed us to observe that in the non-MACE group (n = 76) most patients were placed in the low-risk category with a HEART score of 0–3 (48/76 patients, 63.2% of the non-MACE group) or in the intermediate-risk category with a HEART score of 4–6 (27/76 patients, 35.5%).

In contrast, in the MACE group, 18 patients out of 26 (69.2%) were included in the high-risk group with a HEART score of 7–10 and 8 patients (30.8%) were assigned to the intermediate-risk group with a HEART score of 4–6.

These results were confirmed by the statistical analysis comparing the HEART score with troponin measurements at the various sampling times. (Figure 1).

The data on the risk of developing MACEs from the study for the low-, intermediate- and high-risk HEART score categories were 0% (0/48 patients who did not experience cardiovascular events), 28.5% (10/35 patients who developed MACE) and 84.2% (16/19 patients), respectively.

In the first study of the HEART score, Six et al. found that the three categories of increasing HEART scores had a 2.5%, 20.3% and 72.7% risk of developing cardiovascular endpoints, respectively [11].

In a subsequent HEART score validation study conducted by Backus et al. [16], adverse cardiovascular events at 6 weeks occurred for 1.7% in the low-risk HEART score group, 16.6% in the intermediate-risk HEART score group, and 50.1% in the high-risk HEART score group.

From the comparison with the data in the literature (Table 6), the results of our study in question are partially in agreement, especially in the categories of low- and intermediate-risk HEART scores. The largest discrepancy is observed in the high-risk HEART score group, probably due to the use of high-sensitivity troponin assays, which were not available at the time the earlier studies were conducted. With regard to the low-risk group, the same conclusions have been reported in several studies, including more recent. In particular, in the retrospective study by Vaskas (2025) involving more than 10,000 patients presenting to the ED with chest pain, similar findings were observed (30-day MACEs in patients with a HEART score <3: 1.6% vs. 0.6%, *p* < 0.001; and in patients with a HEART score >6: 6.6% vs. 1.6%, *p* < 0.001) [29,30].

Different conclusions were reported by Ragip (2025) in his study of 560 patients, which showed a 30-day MACE rate of >4% in the low-risk HEART score group, and by Janese (2025) in a cohort of 1284 patients, who found a 90-day MACE rate of 4.4% in the same risk category [31,32]. One reason for these differing results may be attributed, for example, to the proportion of female subjects within the study population, in addition to the issue of inter-operator reliability [33,34].

### 4.3. TIMI Risk Score

Considering the stratification of the TIMI risk score into the three risk groups (TIMI risk score 0–2 are patients considered low risk, TIMI risk score 3–5 are patients considered intermediate risk, TIMI risk score 6–7 are patients considered high risk), the analysis of the study sample showed that 68 patients out of 76 (89.5%) who did not develop MACEs were placed in the low-risk group, with the remaining 8/76 (10.5%) included in the intermediate-risk group. In contrast, most patients who developed 30-day MACEs were included in the intermediate-risk group with 19 out of 26 patients (73.1%); 5 patients were in the low-risk group (19.2%) and 2 patients in the high-risk group (7.7%).

As regards the TIMI score, the percentage of MACEs developed by patients at 30 days for each point of the score was calculated and compared with the MACEs predicted by the TIMI risk score, as shown in the study by Antman et al. [17] (Table 6).

From the comparison, it was possible to observe the discrepancy in absolute terms of the percentages of MACEs for each point of the score; however, it is noted that even in our study sample, as the TIMI score increases, there is an increased risk of developing adverse cardiovascular events.

The most significant differences were observed in patients with TIMI risk scores from 2 to 6, in particular the 11 patients with TIMI score 2, 36.4% of whom (4/11 patients) developed MACEs. This is noteworthy as patients with TIMI risk score 2 are considered to be at low risk.

Our study highlights a tendency of the TIMI risk score to underestimate the risk of developing adverse cardiovascular events, especially in low-risk categories, as evidenced in the original study by Antman et al., which found a 4.7% risk of developing MACEs even for patients classified with TIMI score 0 [17].

This limit of the TIMI risk score was confirmed by statistical analysis that compared the TIMI score with the hs-cTnI measurements at various times. In particular, in the MACE group stratified by the TIMI risk score, it was possible to observe the presence of patients with troponin values from 2000 to 6000 pg/mL classified in the TIMI risk score group 0–2 considered at low risk.

### 4.4. Comparison of the Three Clinical Scores

Comparing the clinical scores considered in our study, it was shown that there are differences in terms of prognostic reliability. This estimate could derive from the way in which the three scores were achieved.

The CPS is a chest pain clinical assessment tool that is affected by the subjectivity of the patient who describes the characteristics of their pain and the subjectivity of the operator who collects this information.

Our study showed that no patient with MACEs at 30 days had a CPS < 4; this could have important clinical findings since individuals with CPS < 4 (low risk) could be discharged early after a short period of observation and further first-level examinations (ECG and normal cardiac biomarkers) negative for ACS. The use of CPS would also apply to low-risk patients with CPS ≥ 4, who would be referred to a short observation period since it has been noted that 20% of these low-risk patients are likely to develop coronary artery disease [15,20].

These data are confirmed by the comparative analysis of CPS with hs-cTn values in the group of non-MACE patients since some individuals with CPS ≥ 4 showed non-significant increases in troponin values from T0 to T2, which, however, may suggest further investigation.

Furthermore, considering the correlation matrix expressed in Figure 1, it was noted that the only positive correlation index (value = 0.03) of the CPS was at time T0, suggesting that this is the most suitable time to apply the CPS, i.e., at the time of the patient’s admission and triage.

As highlighted by our study, the TIMI risk score correctly identifies most subjects at low risk of MACEs; however, a small proportion of patients classified as low risk (TIMI 0–2) still experienced events. This limits the usefulness of the score in the management of patients with suspected low-risk ACS who might otherwise be considered for early discharge. This limitation may stem from the fact that the TIMI risk score was originally developed in a cohort of patients with confirmed NSTEMI/UA, and therefore expresses its best predictive performance in hospitalized subjects with established ACS, where it provides support for clinical management rather than in patients assessed in the pre-diagnostic phase in triage [12,35,36,37,38].

This last consideration was supported by the analysis of the correlation matrix for the TIMI risk score in the MACE group (Figure 1C), in which the correlation coefficients at T0 (r = 0.13), T1 (r = 0.27), and T2 (r = 0.17) showed that the TIMI risk score correlates more strongly with T1, likely corresponding to the time when the diagnosis of NSTEMI/UA was established.

The comparison of the ROC curves, together with the univariate and multivariate analyses, ultimately indicates that the score most helpful to emergency physicians in assessing the short-term risk of patients presenting with chest pain is the HEART score.

The results of our study showed that the HEART score allows patients to be correctly placed in the different risk groups and consequently offers valuable support for the decision of early discharge for low-risk patients and immediate recourse to invasive maneuvers for high-risk patients. The confirmation of these data was obtained with the correlation matrix of the HEART score in the group of MACE patients (Figure 1B), in which it is observed that the correlation indices at times T0 (r = 0.40), T1 (r = 0.31) and T2 (r = 0.52) are mostly overlapping, indicating an excellent prognostic reliability of the HEART score both in the initial phase and in the advanced phase of management of the patient with chest pain. The improved management of patients at low risk of MACE with the HEART score is due to the fact that the score was designed based on a sample of patients with general chest pain [38].

Ultimately, the HEART score has a higher predictive power than the CPS and the TIMI risk score as underlined by the comparison of the AUC values of the ROC curves calculated for the three scores shown in Table 2. This comparative analysis confirms similar data present in the literature as evidenced by the study by Poldervaart et al. [37] showing an AUC of 0.86 (95% CI: 0.84–0.88) for the HEART score compared to an AUC of 0.80 (95% CI: 0.78–0.83) for the TIMI risk score.

The DeLong test for the comparison of ROC curves also showed the superiority of the HEART and TIMI scores over the CPS and confirmed that the HEART score performs better than the TIMI score. The multivariate analysis likewise showed that hs-cTnI measurement at the three assessed time points represents a key parameter in determining the outcome of patients presenting with chest pain.

Moreover, we evaluated the potential predictive value of the HEART score in combination with the CPS; based on the data obtained, we can state that no additional predictive power is gained. Therefore, we consider the use of the CPS by nursing staff during the triage phase in the emergency department to be particularly relevant.

### 4.5. Limitations

The present study has several limitations that should be considered when interpreting the results. First, the sample size was relatively small (n = 102), as this was a pilot study conducted over a limited enrollment period of seven months. The number of eligible patients was further reduced by the application of strict inclusion criteria (age ≥18 years, atraumatic chest pain, informed consent) and by the exclusion of individuals whose initial presentation was not confirmed as chest pain upon medical re-evaluation, as well as those lost to follow-up. Second, the requirement for a complete 30-day follow-up inevitably contributed to the reduced sample size, since patients lost to follow-up were excluded from the analysis. Although this approach strengthens the reliability of outcome assessment, it may have introduced selection bias and reduced the external validity of the findings. Third, as a single-center observational study, the results may reflect the specific clinical practices, patient population, and organizational characteristics of our emergency department, and therefore may not be fully generalizable to other settings or healthcare systems. Moreover, the study was conducted in a high-volume emergency department, where overcrowding, time constraints, and workload may influence data collection, limiting the possibility of recruiting a larger sample within the available timeframe. Finally, the study should be regarded as an exploratory or pilot analysis, intended to provide an initial comparison between the chest pain score, HEART score, and TIMI risk score in real-world ED conditions. The limited number of patients does not allow definitive conclusions; however, the findings support the prognostic value of the HEART score in particular and highlight the need for larger, multi-center studies with longer follow-up to confirm and expand these results.

## 5. Conclusions

The management of patients presenting to the ED with chest pain remains a major clinical and organizational challenge due to the high volume of admissions and the need for rapid and accurate risk stratification. Our findings highlight the practical value of integrating clinical scoring systems into routine ED assessment to support timely decision-making.

Among the tools evaluated, the HEART score demonstrated the highest prognostic accuracy across the early and advanced phases of patient evaluation, making it a particularly useful instrument for guiding clinical decisions and identifying patients who may require closer monitoring or early cardiology consultation. The chest pain score proved especially helpful at triage, offering a rapid and structured approach to characterize suspected cardiac chest pain and to safely identify low-risk patients who may benefit from expedited management pathways. Conversely, the TIMI risk score showed its greatest utility in patients with confirmed ACS, supporting risk stratification and therapeutic planning after diagnosis.

From a clinical standpoint, these results reinforce the importance of adopting a score-based approach to streamline ED workflows, reduce unnecessary admissions, and improve the allocation of diagnostic and therapeutic resources. Although limited by the single-center design and sample size, this study provides additional evidence supporting the complementary use of these scores in different phases of ED management. Larger multi-center studies are warranted to confirm these findings and to further refine practical algorithms for chest pain evaluation.

## Figures and Tables

**Table 1:** Main characteristics of the study sample. The MACE group is older and presents a greater cardiovascular burden. Clinical suspicion shows a stronger effect in the MACE group.

	Total Patients (nr 102)	Group No MACE (nr 76)	Group MACE (nr 26)	*p*-Value
Baseline demographics				
Mean age (±SD)	59.4 ± 15.2	57.13 ± 15.6	67.3 ± 10.83	0.0005 **
Male sex (%)	70 (68.6)	48 (63.2)	22 (84.6)	0.0512 *
Associated symptoms				
Dyspnea (%)	37 (36.3)	27 (35.5)	10 (38.5)	0.8162 *
Nausea (%)	7 (6.9)	4 (5.3)	3 (11.5)	0.3671 *
Diaphoresis (%)	31 (30.4)	22 (28.9)	9 (34.6)	0.6259 *
Medical history				
Highly suspicious (%)	25 (24.5)	3 (3.9)	22 (84.6)	<0.0001 *
Moderately suspicious (%)	33 (32.4)	29 (38.2)	4 (15.4)	0.0504 *
Slightly suspicious (%)	44 (43.1)	44 (57.9)	0 (0)	<0.001 *
Cardiovascular risk factors				
High blood pressure (%)	52 (50.9)	32 (42.1)	20 (76.9)	0.0029 *
Hypercolesterolemia (%)	54 (52.9)	33 (43.4)	21 (80.7)	0.0012 *
Diabetes mellitus (%)	22 (21.6)	13 (17.1)	9 (34.6)	0.0949 *
Smoking (%)	29 (28.4)	21 (27.66)	8 (30.7)	0.8035 *
Cardiovascolar family history (%)	17 (16.7)	13 (17.1)	4 (15.4)	1.00 *
BMI ≥ 25 (%)	59 (57.8)	43 (56.6)	16 (61.5)	0.8185 *
Previous cardiovascular disease				
Coronary artery disease (%)	16 (15.7)	4 (5.3)	12 (46.1)	<0.001 *
Previous ACS (%)	10 (9.8)	4 (5.3)	6 (23.1)	0.0163 *
Carotid atherosclerosis (%)	6 (5.9)	4 (5.3)	2(7.7)	0.6433 *
Stroke (%)	2 (2)	1 (1.3)	1 (3.8)	0.4467 *
Peripheral artery disease (%)	1 (1)	0 (0)	1 (3.8)	0.2549 *
High-sensitivity troponin at time 0				
Normal (%)	88 (86.3)	76 (100)	12 (46.1)	
Higher than cut-off (%)	14 (13.7)	0 (0)	14 (53.9)	
Median TNI (IQR)	102	4 (2.6–7.45)	59.5 (13.9–362.9)	<0.000 **
CPS				
<4 (%)	24 (23.5)	24 (31.6)	0 (0)	
≥4 (%)	78 (76.5)	52 (68.4)	26 (100)	
Total CPS (IQR)	102	7.5 (4–9)	9 (8.5–12.5)	<0.001 **
HEART score				
0–3 (%)	48 (47.1)	48 (63.2)	0 (0)	
4–6 (%)	35 (34.3)	27 (35.5)	8 (30.8)	
7–10 (%)	19 (18.6)	1 (1.3)	18 (69.2)	
Median HEART score (IQR)	102	3 (2–4)	7 (6–8)	<0.0001 **
TIMI score				
0–2 (%)	73 (71.6)	68 (89.5)	5 (19.2)	
3–5 (%)	27 (26.5)	8 (10.5)	19 (73.1)	
6–7 (%)	2 (1.9)	0 (0)	2 (7.7)	
Median TIMI score (IQR)	102	0.5 (0–1)	4 (3–4.75)	*p* < 0.0001 **

* Fisher’s exact test, ** Mann–Whitney U test.

**Table 2:** AUC values of the ROC curves for CPS, HEART score, and TIMI risk score.

	**AUC Values of ROC Curves**
**Chest pain score**	0.8312 (95% CI: 0.7469–0.9155)
**HEART score**	0.9757 (95% CI: 0.9523–0.9991)
**TIMI risk score**	0.9378 (95% CI: 0.8926–0.9829)

**Table 3:** DeLong’s test (pairwise) reporting the Z score and *p*-value.

Comparison	Difference AUC	Z	*p*-Value
HEART vs. TIMI	0.038	1.37	0.17
HEART vs. CPS	0.144	3.25	0.001
TIMI vs. CPS	0.106	2.43	0.01

**Table 4:** Univariate analysis in which age is expressed as OR for +10 years, scores are expressed as OR for 1-point increase, and hs-cTnI is expressed as OR for log10 (pg/mL).

Variable	MACEs	No MACEs	OR	IC 95%	*p*-Value
CPS	9 (8.5–12.5)	7.5 (4–9)	1.487	1.221–1.811	<0.001
HEART score	7 (6–8)	3 (2–4)	3.525	2.039–6.094	<0.0001
TIMI score	4 (3–4.75)	0.5 (0–1)	3.642	2.181–6.082	<0.0001
Male sex	22/70	48/70	2.90	0.903–9.315	0.111
Age	67.3 ± 10.8	57.13 ± 15.6	1.053	1.015–1.093	0.007
hs-cTnI T0	59.5 (13.95–562.95)	4 (2.6–7.45)	7.84	3.03–20.27	<0.001
hs-cTnI T1	570.8 (58.85–2796.8)	4.9 (2.9–8.65)	6.15	(2.60–14.55)	<0.001
hs-cTnI T2	2537.55 (410.4–11,350.85)	9.75 (3.93–41.38)	12.02	(1.28–112.82)	0.03

**Table 5:** Comparison between HEART only and HEART + CPS.

Model	AUC	AIC
HEART only	0.955	49.99
HEART + CPS	0.956	51.40

Akaike Information Criterion (AIC).

**Table 6:** Comparison between observed MACEs in the study sample and predicted MACEs from the literature using the TIMI score.

TIMI Score (Number of Patients)	Percentage of 30-Day MACEs in the Study Sample (Number of Patients)	Percentage of MACEs Predicted by Antman et al. [17]
0 (38)	0% (0)	4.7%
1 (24)	4.2% (1)	4.7%
2 (11)	36.4% (4)	8.3%
3 (12)	58.3% (7)	13.2%
4 (11)	72.7% (8)	19.9%
5 (4)	100% (4)	26.2%
6 (2)	100% (2)	40.9%
7 (0)	0% (0)	40.9%

## Data Availability

The data presented in this study are available on request from the corresponding author. The data are not publicly available due to the privacy reasons (sensitive data). This statement was unanimously agreed upon by all the authors.
